# Supplementary material for: Patterns of Intron Gain and Loss in Fungi
Source: PLoS Biol. 2004 Nov 30;2(12):e422. doi: 10.1371/journal.pbio.0020422 (PMC532390; doi:10.1371/journal.pbio.0020422)
Supplement: Table S1 — Also available at http://genes.mit.edu/NielsenEtAl/. (4.3 MB ZIP). [file pbio.0020422.st001.zip › NielsenEtAl/html/736.html]

AN6978.1.NCU05513.1.MG00408.1.FG08173.1


```
 CLUSTAL W (1.82) Multiple Sequence Alignments - Introns Inserted


Sequence 1: NCU05513.1	618 aa
Sequence 2: MG00408.1	669 aa
Sequence 3: FG08173.1	594 aa
Sequence 4: AN6978.1	533 aa
Alignment Length: 687 aa
Number Identitical Residues: 185 aa
Alignment Score (without introns) 10570


MG00408.1 	MPPKKAAATKAAAAPAAKKAAPAKKSAGVTKTTAPAKKTAAAAKPKAAPAKAAPAKAAPV
NCU05513.1	MPPKKAAATQTAKENAPAAKTATKKTK--TTTTVTKKTTAKVAKKDEEEPKAAPKTAATK
FG08173.1 	MAPR----------AAVARAAATKKST--------TTTTAAAAKKDAKPTKAAAKKAEP-
AN6978.1  	MPPK---------GASKPAARATTTSK--------KKTATTASTTKNSRGRAAATTKTT-
          	*.*:           :     .:..:          ..:: .:. .    :**. .  . 

MG00408.1 	KAAPAKAAATKAAPKKRKVAEEPEPEKEEEEEVEQAQDEDSEEEEEKPAKGSRKSAPPAT
NCU05513.1	KTAAPKAAAKKAPAPKKTAAPKAAAKK---------------------APAPKKAAAPKK
FG08173.1 	--------AINGASKKRTRAEEQEVEE---------------------DEEEEVTRKTKK
AN6978.1  	--------VKSTATRKTTKADEVGATK--------------------------KTAAPAK
          	        . . .. * . * :    :                           :  . .

MG00408.1 	KKRSTPVPAPKRAASAQPKKAKAAATKKKTPAPEADKENADPEPKVTGASSPTKRKREDT
NCU05513.1	TAAAKKAAAAAKDDSDKENSVAGAKRKREEDEEEEEQEELADEKQVNGTRPMVKR-----
FG08173.1 	TKTTERVVKTATKPAAKAKTTKPAASKAKAKATSEEPEEKKPVAKAKTAKP---------
AN6978.1  	KRRADAEDAETSRSTKRARVVKPAAAK---------------------------------
          	.  :          : : . .  *  *                                 

MG00408.1 	VEPSHDRGAAKDESEAEDKPAPEREIKQPRPVKRVRTAAPVSKVKIGAKINNAPDAVLDV
NCU05513.1	---------AKTDEDEAAKPAPKKAKAAPKPKPEPK------KKPLGKVINEAPTKVMDI
FG08173.1 	--------------AAAKKAEPKKKTSFPG---------------IGKKINEAPTKILDV
AN6978.1  	-------------------PRPK------------------------VVINNAPTAKLNV
          	                   . *:                          **:**   :::

MG00408.1 	FVFGEGSSGELGLGSMKYENKKPIDVKRPRINHNLSADKVGVVQLDCGGMHAIALTKDNK
NCU05513.1	FVFGEGTSGELGLGSLKYDGKKPIDVKRPRINHNLS----GIVQVACGGMHVAALTKDNK
FG08173.1 	YVFGEGSSGELGLGSKRINNKKPIDVKRPRLNDNLAAATTGVVQISCGGMHAVALTHDNK
AN6978.1  	YVCGEGSSGELGLG----VGKNVIDVKRPRLNPHLLPDDVGVVQVAVGGMHCVALTHDNK
          	:* ***:*******     .*: *******:* :* .   *:**:  ****  ***:***

MG00408.1 	VLTWGVNDQGALGRDTTWDGGLRDADAEDS--DSDSEDGEDSGLNPVESNPAEIDMSDVA
NCU05513.1	ILTWGVNDQGALGRDTTWDGGLRDADAE-----SDSEDEDDTGLNPKESTPGEIDTTDVP
FG08173.1 	ILTWGVNDQGALGRDTTWDGGLRDMDSAE---DSDSDDEDDSGINPKESTPTAVDDEFFA
AN6978.1  	VLTWGVNDQGALGRDTTWEGGYKDMDNRDSDSDSDSDSDDNPDLNPHECTPTAIPSSAFP
          	:*****************:** :* *  .:.:.***:. ::..:** *..*  :    ..

MG00408.1 	EGTRFVQVAASDSASFALTEDGRVYGWGTFR0ASDGVLGFTPTIKIQKFPTLLAEPKKVV
NCU05513.1	EGTVWVQVVASDSATFALTATGQVYGWGTFR0SNEGILGFTRNVLIQKTPALITQLSKIK
FG08173.1 	PGTKFVQVVASDSASFTLTEDGRVYGWGTFR~SSDGILGFSETIKIQPTPLLIKDLKNIK
AN6978.1  	HGTVIVEVAAGDSSSFALTDEGQVYGWGTFR0SNDGILGFDAKTKVQTTPKLLPDLKKIK
          	 **  *:*.*.**::*:**  *:******** :.:*:***  .  :*  * *: : .:: 

MG00408.1 	QIAAGSNHAMALDSAGKLYTWGCPEQNQLGRRCVQRELLASALRPGGVGLKRG-VKVVKI
NCU05513.1	QLAVGSNHVLALDEKNKVFAWGAGQQAQLARRLLERDENG-ALFPASIGTLPGRAKAAKL
FG08173.1 	ALSAGSNHILALDHKGNVVAWGCGQQNQLGRRIIERNKMS-SLIPQGVGLPRG--KIAKI
AN6978.1  	HLVCGDNHVLALNDKGAVLSWGSGQQNQLGRRIIERNKLN-GLQPREFGLPKG---IVHI
          	 :  *.** :**:  . : :**. :* **.** ::*:    .* *  .*   *    .::

MG00408.1 	SCGSYHSFAVDKEGGVYTWGLNNFGQLAIEQDAGDSDAAVLTAVKVESLMDYKIVDIAGG
NCU05513.1	ACGSYHCFIIDTKGRVIGWGLNNYAELGIEAEAGTDGGFILKPQLVDSLKEHNIVDIAGG
FG08173.1 	ACGSYHSFAIDKEGVVYGWGLNNFGEIGVESNAGEDDAVILRPAKLTYLDDYNITEIDGG
AN6978.1  	GAGAFHSFAVHQSGKVFAWGLNSFGETGIRENAGDSEAAIVHPTVVDSLSKKNVTQICGG
          	..*::*.* :. .* *  ****.:.: .:. :** . . :: .  :  * . ::.:* **

MG00408.1 	EHHSLACTDDGRLLVWGRIDGHQTGLPFDSFTEENAIYDDYKKPRILKTPTVIPG1LP-K
NCU05513.1	EHHSLATNDKGELLTWGRIDGHQVGHPSGSFTEENTIFDEKDKPRILVQPAVVPD~IEGK
FG08173.1 	EHHSLACSDKGDLLTWGRVDGYQVGFEFDKLSEENSIYDERGNARILFKPTIQPD1VK-D
AN6978.1  	AHHSIAATQDGECLVWGRLDGYQTGLKIDTLPDDAVIKDERDRPRILIEPTAVPG~IK--
          	 ***:* .:.*  *.***:**:*.*   ..:.::  * *:  ..***  *:  *. :   

MG00408.1 	IVSVAAGTDNSFAITEDGKVYSWGFSINYQTGQGTSDDVEAPTLIDNTAVRDRNITFAGA
NCU05513.1	IVHVAAGTDHSFAITEDGKVYSWGFSANYQTGQGTTDDIETPTLIDNSAIRDRKIIFAGA
FG08173.1 	IVSVAAGTDNNFAISSNGTVYSWGFSSNYQTGQGTIEDIHTPTLIDNTAIRGKKIIGAGA
AN6978.1  	AKAVAAGSDHSIAIDTSGRPWSWGFSATYQTGQGTQDDVEVATVIENTAVRGKSLNWAGG
          	   ****:*:.:**  .*  :***** .******* :*:...*:*:*:*:*.:.:  **.

MG00408.1 	GGQYSMLACHAEKKA--------------
NCU05513.1	GGQYSILGAEPKE----------------
FG08173.1 	GGQYSVLIGEAEDVPQTNGAATNGTDDKA
AN6978.1  	GGQFSVFTEPVEL----------------
          	***:*::    :
```
